# Immunophenotyping beyond the limits of time

**DOI:** 10.1016/j.crmeth.2023.100612

**Published:** 2023-10-25

**Authors:** J. Fréderique de Graaf, Ramon Arens

**Affiliations:** 1 Department of Immunology, Leiden University Medical Center, 2333ZA Leiden, the Netherlands

## Abstract

Immunophenotyping is a powerful approach for deciphering responses of the immune system to drug screening and immunotherapy. In this issue of *Cell Report Methods*, Liechti et al. have advanced this approach by developing a pipeline, which allows high-throughput but still accurate single-cell immunophenotyping in time.

## Main text

Within the last decades, our understanding of the immune system and its responses to medical interventions, e.g., immunotherapy, has grown extensively using immunophenotyping. Examples include the detection of innate and adaptive immune cell subsets associated with either health or disease and determination of the characteristics of disease-specific cells to immunotherapy.^1^^,^^2^ In this respect, flow cytometry has been demonstrated to be an accessible method to perform such valuable immunophenotyping. In the technique, single cells within a population are stained with fluorophore-labeled antibodies that identify cell-surface markers and/or intracellular proteins, such as cytokines or transcription factors. A fluid stream of stained suspended cells is then illuminated by different lasers, resulting in distinct excitation of the respective fluorophores and subsequent detection of their fluorescence by separate detectors (conventional flow cytometry) or a detector array (spectral flow cytometry). The detected fluorescence signal correlates with structural characteristics, e.g., size or granularity and the expression of individual proteins of each cell or event measured. Potential fluorescence spill-over is mathematically corrected, referred to as compensation or unmixing. Thus, even if fluorochrome spectra overlap, markers can be distinguished allowing the detection of more fluorophores per sample. The maximum number of detectable fluorochromes within one sample is approximately 18 fluorophores for conventional flow cytometers. New detection methods based on full spectral analysis have made it possible to measure up to 40–50 fluorophores per sample, resulting in advanced high-dimensional immunophenotyping.^3^ The latter approach comes even close to the number of markers measured by mass cytometry,^4^ which is an alternative multi-parametric cytometry method that relies on the detection of antibodies labeled with heavy-metal isotopes. Mass cytometry, however, is less suited for high-throughput analysis of many cells given that the acquisition time for a certain number of cells is much longer compared to flow cytometry.

Several studies have demonstrated the value and possibilities of high-dimensional flow cytometry in both infectious disease^5^ and cancer.^1^^,^^2^ However, large cohort studies or time-connected studies have been challenging due to the length of the measurements of the samples or the time intervals. For instance, changes in signal intensity can occur during long-lasting measurements leading to inter-experimental variation. Moreover, data acquisition at different time points can also be biased by technical and experimental variation leading to inter-assay variation in compensation or in differential fluorescent intensity.^6^ Therefore, there is a clear urgency for methods to implement quality controls allowing performance tracking in order to maintain consistent signal and data generation.

**Figure 1:**
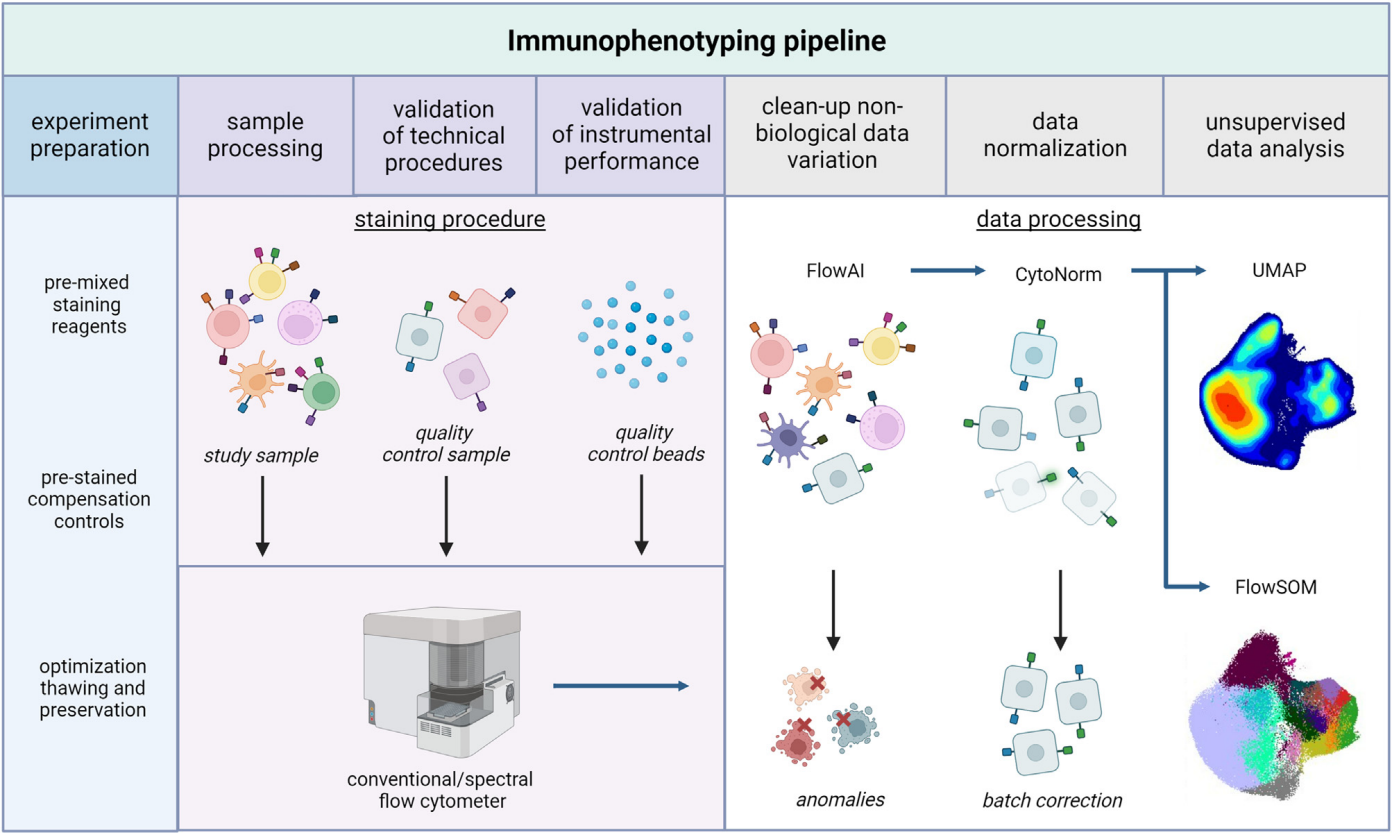
A pipeline for immunophenotyping in large cohorts or time-connected studies using high-dimensional flow cytometry and unsupervised data analysis Schematic representation of sample processing for precise, accurate, and consistent data acquisition using either conventional or spectral(-enabled) flow cytometry followed by unsupervised data analysis. The pipeline consists of steps that optimize sample preparation protocols, takes into consideration technical variations, validates instrumental performance, and removes low-quality events and inter-assay differences ultimately performing unsupervised data analysis via uniform manifold approximation and projection (UMAP) or FlowSOM. Figure created using BioRender (https://biorender.com).

Here, we preview a study by Liechti et al. revealing age-associated immune dynamics in 2,300 individuals based on the analysis of 3,357 samples using three different antibody panels of 28 fluorophores, each defining either B cells and myeloid cells, T and NK cells, or T cell function.^7^ The samples were processed within 6 months via 19 separate acquisitions. Optimization of staining procedures included premixing and storage of reagent cocktails, assessment of pre-stained compensation controls comparing bead- versus cell-based single stains, cell resting, and viability assessments (Figure 1). Technical and experimental variation was extensively controlled by stringent instrument standardization and quality controls within staining procedures followed exclusion of anomalies in the acquired data and unsupervised data analysis, including FlowAI, CytoNorm, FlowSOM, and uniform manifold approximation and projection (UMAP).

Increased numbers of fluorophores complicate matters in filtering out non-biological data variation in spectral flow cytometry datasets compared to conventional flow cytometers. As a result, defining variation between measurements is necessary to ensure precise, accurate, and consistent data acquisition. Using conventional flow cytometry, several studies have previously accurately quantified different immune phenotypes in longitudinal cohort studies by explaining their non-biological variation based on correlation between intra-individual and technical variation.^8^^,^^9^ However, these datasets were acquired with smaller panels consisting of <18 fluorophores per panel. The accessibility to larger panels using spectral(-enabled) analyzers could improve in-depth phenotyping but increase the complexity of these correlations. To determine measurement precision and hence the data consistency and inter- and intra-individual data variation, verified quality controls were implemented for both instrument standardization and experimental procedures in the work by Liechti et al. (Figure 1).

Instrumental standardization is required for consistent performances over time and between instruments.^6^^,^^10^ The technical variation was quantified by fluorochrome beads where results demonstrated the variance of spectral compensation values and spill-over spreading between the 19 experiments to be low and assured the feasibility of the same gating strategy over all samples. High precision of cell frequency measurements was accounted for by using a pre-defined quality control (QC) sample. The data retrieved from these QC samples through 19 experiments and three panels demonstrated that most datasets aligned along the Poisson distribution, suggesting a low Poisson counting error and thus high measurement precision.

High measurement precision was partly obtained by the extensive pre-processing of data including quality checks, such as cell viability and cytokine production, but also with the clean-up of non-biological data variation induced by events in flow rate or signal acquisition and outliers in the dynamic range using FlowAI (Figure 1). This resulted in the removal of low-quality events from all samples followed by manual spectral overlap corrections. The immune phenotyping pipeline was further extended by unsupervised data exploration, which is considered less biased compared to manual gating (Figure 1). However, unsupervised data algorithms are affected by spill-over spreading, low-quality samples, and inter-assay differences.^11^ To overcome batch effects, data were first normalized using CytoNorm, which was trained on the QC samples from the 19 different experiments. Subsequently, dimension reduction algorithms, including UMAP and FlowSOM, were used to interpret the data in addition to manual gating. The analysis demonstrated the high reproducibility of the QC samples, which appeared in a small group within the UMAP, whereas all other data were clustered on age and cytomegalovirus (CMV) serostatus but, interestingly enough, not gender.

The high measurement precision allowed the longitudinal assessment of intra-individual immune changes in 158 individuals over a period of more than 5–10 years. Intra-individual variation was defined as the variation between a primary sample and samples required after 2, 4, or 6 years or longer and was assessed by surface markers and cytokines on different immune subsets, including CD4^+^, CD8^+^, and γδ^+^ T cells. The data indicated that the human immune system has a high temporal stability in which intra-individual immune variation was smaller than inter-individual variation between individuals. This included the stability of the expression of several immune receptors, including CD28 and CD127 on CD4^+^ T cells or IL21R on mature B cells, and also T cell effector functions for at least a period of 10 years. This is in line with previously published results demonstrating a wide inter-individual variation, which is dependent on age, cohabitation, and genetic background.^8^^,^^9^

Further analysis of the data was based on FlowSOM, an unsupervised technique for cell meta clustering and dimension reduction in which subjectiveness of manual analysis is avoided.^12^ Meta clustering of both the B and myeloid cell panel and the T and NK cell panel indicated 40 different population of which several were affected by age. These populations included naive T cells, mucosal-associated invariant T (MAIT) cells, Vγ9^+^Vδ2^+^ T cells, and memory B cells (IgG^+^, IgD^+^IgM^dim^CD27^+^, and/or CD11c^+^), while HLA-DR^+^ transitional memory CD4^+^ T cells increased with age. In addition to age-dependent phenotyping, meta clustering of the data indicated a specific subset of stem cell-like T cells, which was considerably increased in a small group of individuals compared to the total cohort. SNP analysis of available genomic data confirmed a rare mutation leading to a splicing defect from CD45RA to CD45RO resulting in increased levels of either CD4^+^ or CD8^+^ stem cell-like CCR7^+/dim^CD27^+^CD28^+^CD45RA^+^CD95^+^ T cells, whereas CD45RA^−^CD95^+^ memory cells were reduced. Overall, these data demonstrate the possibilities of highly accurate and precise measurements with spectral analyzers in defining immune phenotypes through time.

Taken together, Liechti and colleagues have developed a pipeline to perform accurate and precise measurements on spectral(-enabled) analyzers.^7^ Using the pipeline, the authors obtain results that are comparable to previous studies using conventional flow cytometers. The described pipeline allows the standardization of sample processing and analysis of samples measured by spectral flow cytometry for immunophenotyping, including several verified controls (Figure 1). The verification method was based on the Poisson distribution of the included QC samples and demonstrates this to be an accessible method. Taken together with the extensive data analysis, this work can be used as a guideline for advanced immunophenotyping on spectral flow cytometers.
